# Accountable Care Organization Efficiency on Entry and Shared Savings Bonuses

**DOI:** 10.1001/jamanetworkopen.2026.0166

**Published:** 2026-02-26

**Authors:** Arnav Srivastava, Addison Shay, Samuel R. Kaufman, Xiu Liu, Avinash Maganty, Mary K. Oerline, Paula A. Guro, Dawson Hill, Christopher Dall, Kassem S. Faraj, Meiling Ying, Vahakn B. Shahinian, Brent K. Hollenbeck

**Affiliations:** 1 Dow Division of Health Services Research, Department of Urology, University of Michigan, Ann Arbor; 2 Department of Urology, Massachusetts General Hospital, Boston; 3 Department of Foundations of Medicine, New York University Grossman Long Island School of Medicine, New York, New York

## Abstract

**Importance:**

Spending benchmarks in the Medicare Shared Savings Program previously only considered an accountable care organization’s (ACO) historical spending, potentially disadvantaging efficient organizations in favor of inefficient ones. To more sustainably reward efficient ACOs, benchmark calculation has evolved, such as the incorporation of average regional spending in 2017, but how benchmarking policy, and its changes, have affected the financial performance of ACOs across the efficiency spectrum remains unclear.

**Objective:**

To measure the association between ACO efficiency on entry (ie, the ratio of observed to expected spending) with earning a shared savings bonus.

**Design, Setting, and Participants:**

This cross-sectional study used a 20% national sample of Medicare claims to identify ACOs that participated in the Shared Savings Program for a minimum of 4 years between January 2013 and December 2020. ACOs were sorted in quartiles based on their observed to expected spending ratio in their first year of participants. Data analysis was conducted from July 2024 to May 2025.

**Exposure:**

Quartiles of ACO efficiency with a higher spending ratio denoting lower efficiency.

**Main Outcomes and Measures:**

The primary outcome was receipt of a shared savings bonus in the second, third, and fourth agreement years. Multivariable logistic regression was used to estimate the association between ACO efficiency quartiles and earning a bonus and how the regional benchmark adjustment in 2017 affected this association across measured agreement years.

**Results:**

Across 402 ACOs, the median (IQR) spending ratio was 1.000 (0.993- 1.005). After adjustment, the most efficient ACOs (ie, lowest quartile of the spending ratio) had an increased probability of earning a bonus from 24.4% (95% CI, 15.3%-33.4%) to 45.2% (95% CI, 35.4%-55.0%) after the 2017 introduction of the regional benchmark adjustment. However, the least efficient ACOs (ie, top quartile of the spending ratio) were significantly more likely to earn bonuses prior to (43.8%; 95% CI, 33.7%-53.9%) and after (60.7%; 95% CI, 51.3%-70.1%) the benchmarking change, without evidence that this gap narrowed.

**Conclusions and Relevance:**

In this cross-sectional study, ACOs across the range of efficiency on entry had greater odds of earning bonuses after the introduction of the regional benchmark adjustment in 2017. However, less efficient ACOs had significantly greater odds of earning bonuses compared with more efficient ACOs, before and after the policy change.

## Key Points

**Question:**

Are less efficient accountable care organizations (ACOs) in the Medicare Shared Savings Program more likely to earn bonuses compared with more efficient ones?

**Findings:**

In this cross-sectional study of 402 ACOs between 2013 and 2020, less efficient ACOs on entry more commonly earned bonuses compared with more efficient ACOs. Following the introduction of the regional benchmark adjustment in 2017, there was no significant narrowing of this gap.

**Meaning:**

These findings suggest that the Shared Savings Program preferentially rewards less efficient ACOs compared with efficient ones, even after the introduction of the regional benchmark adjustment.

## Teaser

This cohort study of accountable care organizations in the Medicare Shared Savings Program investigates associations between high and low efficiency with earning a savings bonus.

## Introduction

Accountable care organizations (ACOs) aim to improve value in health care delivery by reducing fee-for-service payment, while maintaining quality. Medicare’s largest ACO program, the Shared Savings Program, includes nearly 500 ACOs that deliver health care to approximately 11 million beneficiaries.^1^ This voluntary program offers organizations the opportunity to share in savings to the Medicare program for achieving quality and spending per beneficiary benchmarks.^2^

Spending benchmarks, the primary determinant of financial success or failure in the Shared Savings Program, have evolved. Initially, the benchmark was solely based on the historical spending trend of an ACO carried forward using national Medicare spending growth. This approach encouraged particularly inefficient organizations (ie, those with higher spending) to participate, as they were seen as having the most excess spending to cut. With historical benchmarking alone, the Shared Savings Program became prone to what is called selection on slopes, where organizations decide to participate according to expected or already achieved reductions in spending.^3^ As a result, inefficient organizations had greater opportunity for reward vs efficient ones, as the latter can continue to reduce spending only to a point.^4^ In 2017, a regional benchmark adjustment was incorporated into benchmark calculations to better encourage participation from efficient organizations. This adjustment additionally includes the average spending in an ACO’s geographic area, likely raising the benchmarks for ACOs that already have relatively lower risk-adjusted spending.^5^ The regional benchmark adjustment appears to have enticed ACOs with lower risk-adjusted spending to participate in the Shared Savings Program.^3^ However, whether this change translates to efficient organizations more easily realizing bonuses over their agreement period is unclear.

We estimated ACO efficiency on entry to the Shared Savings Program by tabulating observed spending against expected spending, adjusting for patient-level characteristics, to create an ACO spending ratio. Given the historical benchmarking arrangement, we hypothesized that ACOs starting with a higher spending ratio (ie, less efficient) would have greater odds of earning a bonus compared with ACOs starting with a lower spending ratio (ie, more efficient). However, we expected that these discrepancies would be mitigated following the introduction of the regional benchmark adjustment in 2017, with the change preferentially helping more efficient ACOs.

## Methods

### Data Sources

We used 2 data sources in this cross-sectional study. First, we used a 20% national sample of fee-for-service Medicare claims. Using the ACO beneficiary-level Research Identifiable File, we identified patients who belonged to an ACO between January 1, 2013, and December 31, 2020, using retrospective attribution. We included only those beneficiaries with continuous enrollment in Medicare Parts A and B in each year and in the preceding year (to capture preexisting comorbidities). We excluded those enrolled in Medicare Advantage plans. Second, we used the Performance Year Financial and Quality Results Public Use File, which contains data at the ACO level.^6^ The file contains financial data pertaining to the Shared Savings Program, such as earned shared savings bonuses. We only examined ACOs that remained in the Shared Savings Program for a minimum of 4 years. This study followed Strengthening the Reporting of Observational Studies in Epidemiology (STROBE) reporting guidelines and was deemed exempt from review and the requirement for informed consent by the University of Michigan institutional review board as patient information was deidentified.

### Observed to Expected Spending: ACO Spending Ratio

Prior work suggests that ACOs with higher spending benchmarks, indicative of lower efficiency, more commonly earn bonuses.^3,7,8^ To more proximally assess ACO efficiency, by incorporating patient mix, we developed an index (ie, ACO spending ratio) measuring observed to expected spending, akin to prior measures of risk-adjusted spending.^3^ We estimated observed spending using price standardized spending, a regionally adjusted measure of global health care utilization derived from Medicare claims data.^9^ The spending for all beneficiaries in a particular ACO-year was summed and its natural log (to account for data skewness) equaled the ACO-year level observed spending.

We postulated that expected spending would be informed by beneficiary-level characteristics (eg, higher spending for older patients with multiple comorbidities compared with younger, healthier patients) and estimated this using Medicare claims data (eTable 1 in Supplement 1). We empirically derived coefficients for beneficiary-level variables (age, sex, race, Hierarchical Condition Category, Medicaid dual eligibility, disability status, and end-stage kidney disease) and performance year using a generalized linear model with an identify link function to estimate expected spending. Hierarchical Condition Categories were informed by *International Classification of Diseases, Ninth Revision* and *International Statistical Classification of Diseases and Related Health Problems, Tenth Revision* diagnosis codes and our analysis accounted for the shift from *International Classification of Diseases, Ninth Revision* to *International Statistical Classification of Diseases and Related Health Problems, Tenth Revision* in October 2015. These coefficients were then applied for each patient to generate beneficiary-level expected spending. The expected spending for each beneficiary in an ACO-year was summed and its natural log equaled ACO-year level expected spending. The ACO spending ratio was calculated by dividing the ACO-year–level observed spending by the ACO-year–level expected spending (both inflation-adjusted). A spending ratio equaling 1.0 implies an ACO spent as much as expected, while a ratio less than 1.0 suggests greater efficiency (ie, spending less than expected). For example, an ACO with an observed spending of $5 million when $8 million was expected according to our adjustment variables would have an ACO spending ratio of 0.97, or ln ($5 million) = 15.42 divided by ln ($8 million) = 15.89. We calculated the ACO spending ratio in the first Shared Savings Program agreement year for each participant, indicating an organization’s efficiency upon program entry and serving as the primary exposure for our study.

### Outcomes

The ACO spending ratio was measured for the first agreement year, and our 2 study outcomes, both measured at the ACO level, were assessed in the following 3 agreement years. The primary outcome was receiving a shared savings bonus, regardless of magnitude, assessed using the ACO Public Use File. We hypothesized that ACOs with a higher spending ratio (ie, less efficient) in the first year would have more room to reduce spending and be associated with greater odds of earning a bonus in the second, third, and fourth agreement years. To measure how the introduction of the regional benchmark adjustment in 2017 impacted the association between receiving a bonus and the ACO spending ratio, we modeled the odds of earning a bonus by calendar year, not agreement year. Finally, in a post hoc analysis, we examined mean bonus per beneficiary across ACO quartiles for the second, third, and fourth agreement years.

### Statistical Analysis

We examined the patient characteristics of ACOs by quartile, according to ACO spending ratio in the first year. Quartile 1 includes ACOs starting with the lowest ratio (ie, most efficient), while quartile 4 includes ACOs starting with the highest ratio (ie, least efficient). To assess the odds of receiving a bonus in the second, third, and fourth years, we used separate logistic regressions. The ACO spending ratio was modeled as a continuous variable (per 0.01 increase).

For each ACO efficiency spending ratio quartile, we used logistic regression to estimate how the regional benchmark adjustment in 2017 was associated with the odds of earning a bonus, across measured agreement years. We modeled calendar years as a fixed effect and created an interaction term with each ACO spending ratio quartile. We then calculated the adjusted percentages of earning a bonus in the preintervention period (2013-2016) and postintervention period (2017-2020). Postulating that regional adjustment would preferentially help the most efficient ACOs, we compared the change between the preintervention and postintervention period by ACO spending ratio quartile, akin to a difference-in-differences framework.

All regression models were adjusted for ACO-level variables (taken from the ACO Public Use File), including the percentage of patients with end-stage kidney disease, disability status, older indviduals (ie, >65 years), and individuals identifying as any race other than White. From these models, the adjusted probabilities were estimated for each quartile of ACOs using postestimation commands. Data were analyzed from July 2024 to May 2025. Analyses were conducted using Stata statistical software version 16.1 (StataCorp). All statistical tests used 2-sided *P* < .05 to define statistical significance.

## Results

### Cohort Characteristics

We identified 402 ACOs that participated in the Shared Savings Program for at least 4 years. The ACO spending ratio in the first agreement year ranged from 0.899 to 1.096, with a median (IQR) of 1.000 (0.993-1.005). ACOs in quartile 1 typically had a lower percentage of older adult patients (mean [SD], 80.70% [9.17%]) and higher percentage of patients with a disability (mean [SD], 15.60% [8.60%]) than ACOs in other quartiles. However, overall, we did not observe any consistent trends in ACO-level patient characteristics with increasing ACO quartile, as illustrated in the Table. When examining patient-level characteristics from Medicare claims data used to calculate expected spending, we noted a slight increase in HCC scores after 2017 in all 4 ACO quartiles, likely representing patients aging within an ACO (eTable 2A and B in Supplement 1).

**Table.** Cohort Characteristics by ACO Spending Ratio Quartile in Baseline Year

Variable	Quartile 1	Quartile 2	Quartile 3	Quartile 4	*P* value^a^
ACO spending ratio, median (IQR)	0.986 (0.977-0.990)	0.997 (0.996-0.998)	1.003 (1.001-1.006)	1.015 (1.011-1.021)	NA
No. of beneficiaries, median (IQR)	14 214 (8849-24 193)	15 031 (10 401-30 070)	12 759 (8831-21 562)	10 173 (7591-15 921)	<.001
Percentage of beneficiaries, mean (SD)					
Elderly	80.70 (9.17)	84.90 (4.93)	84.50 (5.59)	82.50 (7.82)	<.001
Disability	15.60 (8.60)	11.70 (4.63)	12.00 (5.23)	13.90 (7.36)	<.001
End-stage kidney disease	0.95 (0.62)	0.77 (0.39)	0.87 (0.46)	1.09 (0.69)	.01
Any race other than White	15.60 (14.9)	13.40 (10.10)	17.90 (13.90)	20.30 (16.90)	.002
Male sex	43.20 (2.00)	42.70 (1.80)	42.30 (1.70)	42.80 (2.30)	.007

Abbreviations: ACO, accountable care organization; NA, not applicable.

^a^ *P* values were calculated with Kruskal-Wallis test.

### Outcomes

**Figure 1.**
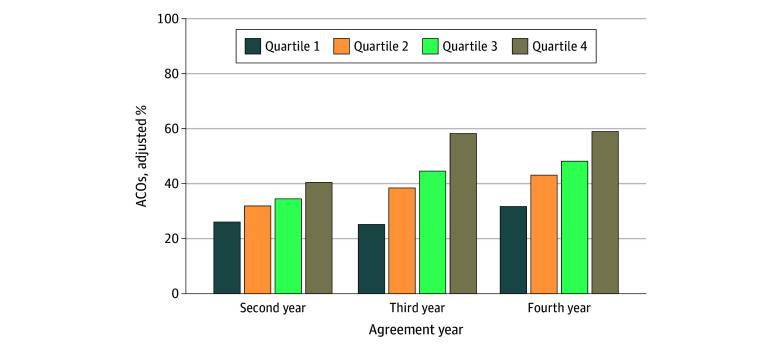
Adjusted Percentage of Accountable Care Organizations (ACOs) Earning a Bonus by ACO Spending Ratio Quartile Percentages are adjusted for percentages of beneficiaries who are elderly, have a disability, and identify as a race other than White. Quartile 1 includes the ACOs with the lowest spending ratio (ie, most efficient) and Quartile 4 includes ACOs with the highest spending ratio (ie, least efficient).

Collectively, 134 ACOs (33%) earned a bonus in the second agreement year, 167 (42%) did so in the third agreement year, and and 183 (46%) did so in the fourth agreement year. Twenty ACOs participated in 2-sided contracts with none paying penalties. After adjusting for ACO-level patient characteristics, an increasing ACO spending ratio (per 0.01 increase) in the first year of participation was associated with greater odds of earning a bonus in the second agreement year (odds ratio, 1.20; 95% CI, 1.03-1.39; *P* = .02). This association was found in the third (odds ratio, 1.50; 95% CI, 1.27-1.77; *P* < .001) and fourth (odds ratio, 1.38; 95% CI, 1.18-1.61; *P* < .001) years (eTables 3-5 in Supplement 1). When estimating adjusted percentages, ACOs in a higher spending ratio quartile were more likely to receive a bonus (Figure 1). This gap was largest in the fourth agreement year, as 59.0% (95% CI, 51.1%-66.9%) of ACOs in quartile 4 received a bonus compared with 31.6% (95% CI, 24.0%-39.3%) among ACOs in quartile 1 (*P* < .001).

**Figure 2.**
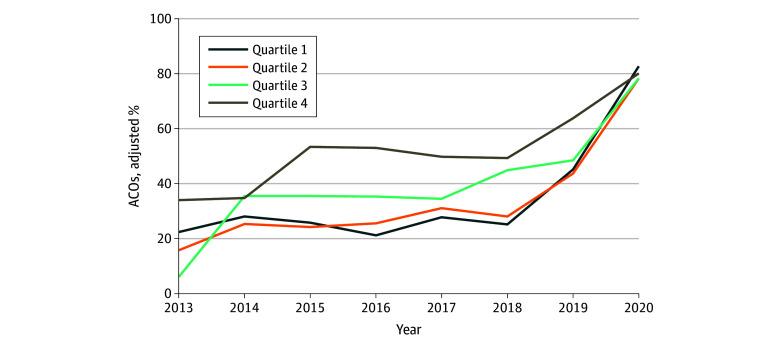
Adjusted Percentage of Accountable Care Organizations (ACOs) Earning a Bonus by Calendar Year and ACO Spending Ratio Quartile Percentages are adjusted for percentages of beneficiaries who are elderly, have a disability, and identify as a race other than White. Quartile 1 includes the ACOs with the lowest spending ratio (ie, most efficient) and Quartile 4 includes ACOs with the highest spending ratio (ie, least efficient).

As Figure 2 illustrates, ACOs in quartile 4 were more likely receive a bonus across the entire study period compared with those in quartile 1 (difference, 21.6 percentage points; 95% CI, 12.6 to 30.7 percentage points). Prior to 2017, the adjusted percentage of ACOs in quartile 4 earning a bonus was 43.8% (95% CI, 33.7% to 53.9%) compared with 24.4% (95% CI, 15.3% to 33.4%) among ACOs in quartile 1. After the introduction of regional benchmark adjustment in 2017, ACOs in quartile 4 (60.7%; 95% CI, 51.3% to 70.1%) and quartile 1 (45.2%; 95% CI, 35.4% to 55.0%) were more likely to earn a bonus compared with the preintervention period. Visually, we observed a convergence in the probability of earning a shared savings in the postintervention period, across quartiles of ACO efficiency. However, on adjusted analysis, we did not observe that ACOs in quartile 1 improved more than those in quartile 4, when comparing the probability of earning a shared savings in the postintervention vs preintervention periods (difference-in-differences estimate, 3.9 percentage points; 95% CI, −14.9 to 22.7 percentage points; *P* = .68).

Finally, ACOs in higher quartiles of the spending ratio (ie, less efficient) earned higher mean bonuses per beneficiary in each agreement year we assessed (eFigure in Supplement 1). For example, in the fourth agreement year, the mean bonus per beneficiary was $226 for quartile 1, $243 for quartile 2, $283 for quartile 3, and $399 for quartile 4.

## Discussion

In this cross-sectional study, our analyses found that ACOs in the Medicare Shared Savings Program that were less efficient in their first year of participation, as denoted by a higher ACO spending rate at entry, had greater odds of earning a bonus compared with their more efficient counterparts. As a result, the least efficient ACOs (ie, top quartile of the ratio) had a 21.6– percentage point likelihood of earning a bonus compared with the most efficient ACOs, over the study period. The introduction of the regional benchmark adjustment appeared to broadly help ACOs, regardless of their efficiency upon entry. However, this did not eliminate the gap in the probability of earning a bonus by preferentially helping efficient ACOs.

This scenario, where less efficient ACOs are preferentially rewarded, illustrates the trade-offs and selective participation implied by the historical benchmarking arrangement. The benchmark setting process directly informs how and which ACOs receive bonuses, critical to the participation of voluntary programs. On one hand, Centers for Medicare & Medicaid Services (CMS) must motivate ACOs with higher risk-adjusted spending to join the Shared Savings Program, which may be achieved through inflated historical benchmarks. This may help CMS achieve large reductions in expenditure, by specifically motivating the least efficient organizations to reduce wasteful spending. As our findings suggest, these organizations may preferentially join with the understanding that they have ample room to reduce low value spending to below benchmark levels and thus receive large bonuses. This reward pattern has been found in early iterations of the Shared Savings Program and among Pioneer ACOs.^10,11^ For such organizations, as indicated in past studies, these higher benchmarks (hazard ratio, 0.80; *P* < .001, per $1000 increase) and the opportunity to earn a bonus (hazard ratio, 0.22; *P* < .001) may make them less likely to leave the Shared Savings Program and encourage additional savings for Medicare.^4^ Furthermore, these cost-saving behaviors may extend to beneficiaries not aligned with an ACO through spillover impacts, potentially amplifying savings in health care broadly.^12^

On the other hand, benchmarking based purely on historical spending creates a ratchet effect for more efficient ACOs that limits the sustainability of their success by making it progressively more difficult to earn bonuses.^3^ As benchmarks get periodically rebased (ie, reset to reflect the most recent level of an ACO’s spending), future potential bonuses are tied to lower, less achievable spending targets.^2,5^ Our analyses found that ACOs with a low ACO spending ratio had lower odds of receiving bonuses. In such a scenario, if 2 ACOs had an identical patient mix, an ACO spending $15 000 per beneficiary with a $20 000 benchmark would be advantaged over an ACO that does not improve relative to its benchmark but continues to spend only $6000 per beneficiary.^6^ This stands to increase the probability that efficient ACOs exit the Shared Savings Program. Here, rather than a useful measure of success, benchmarks may become a problematic design feature of the program as efficient organizations are not sustainably rewarded.^2^ Over time, these challenges may discourage participation in voluntary programs, as long-term success is more difficult to achieve.

Starting in 2017, to address concerns regarding inequities of benchmarks among ACOs in the same region, CMS added the regional benchmark adjustment. Rather than relying only on historical spending, this adjustment also factored in average spending among ACOs within a region. As a result, organizations with relatively lower baseline risk-adjusted spending would receive higher benchmarks than they would under a system that only considered historical spending.^3^ This change attempted to better reward efficient or rapidly improving ACOs. Following this change, ACOs with lower risk-adjusted spending are more likely to participate in the Shared Savings Program, as found in prior work.^3^ Our results do not suggest that these more efficient ACOs are preferentially advantaged compared with less efficient ACOs under this new benchmarking arrangement. However, following the 2017 policy change, we do observe a considerable increase in the odds that efficient ACOs are likely to earn bonuses compared with the preintervention period. Reorienting incentives for ACOs may better motivate changes in practice patterns or investments that may improve care value over an extended time horizon. For example, high-performing ACOs have innovated care delivery with investments in analytics,^2^ telehealth,^13^ and new staffing models (eg, community-based care managers for patients with high-cost chronic illness).^14^ These investments stand to improve patient care, while reducing costs. However, to encourage widespread adoption, organizational leaders would likely want some certainty that future financial windfalls would offset the costs of the upfront investment.^15^ Without aligning incentives for long-term improvement with sustainable financial rewards, the program risks becoming a revolving door for inefficient ACOs that only marginally improve, yet—by virtue of their inflated benchmarks—command substantial bonuses. Akin to what is termed the market for lemons, which describes the unsustainable selection bias seen in insurance markets, preferentially engaging inefficient ACOs may limit the long-term benefits of these voluntary value-based programs.^16,17,18^

Our study underscores a need for benchmarks that motivate and reward the participation of ACOs across the efficiency spectrum. To this end, the regional benchmarking adjustment has added a degree a counterbalance to the historical benchmarking arrangement. However, this need is magnified following the introduction of the Pathways to Success Program in 2019, which increases the financial risk ACOs face and stands to exacerbate selective participation and churn.^19^ One strategy to broadly motivate ACOs is through administrative benchmarks. Administrative benchmarks would have an initial base value tied to historical spending but then unlink the growth (or shrinkage) of the benchmark. Instead, the benchmark’s growth would reflect factors such as policy goals, broad economic indicators (eg, gross domestic product or consumer price index), and anticipated changes in patient mix.^20,21,22^ Policymakers could scale the benchmark’s growth relative to spending to be slower for inefficient ACOs and faster for efficient ACOs, thereby converging benchmarks across ACOs while encouraging broad participation.

### Limitations

This study has limitations. First, we limited our study to ACOs that remained in the Shared Savings Program for at least 4 years. Consequently, we are selecting for ACOs that foresee short-term financial success through the program, as prior work^4^ illustrates earning bonuses is associated with longer participation. This may limit our generalizability to ACOs that leave the program earlier. It may also introduce homogeneity regarding future bonus earning potential, leading to a more conservative estimate regarding the negative impacts of efficiency. Despite this potential underestimation, we still found the financial advantages received by less efficient ACOs. Second, our data only extend to 2020, 3 years after the regional benchmark adjustment was implemented. We may not have the statistical power to demonstrate that this change in benchmarking policy preferentially advantaged efficient ACOs. Despite this, we visually illustrate that the adjusted percentage of earning a shared savings bonus converges across all quartiles by 2020. Third, our study uses a 20% sample of traditional Medicare beneficiaries to estimate ACO efficiency and assumes this would reflect an organization’s overall efficiency (ie, across all beneficiaries). However, our study benefits from a large sample size at the beneficiary level (over 3 million unique patients), mitigating concerns around this assumption. Fourth, when calculating expected spending we risk adjusted using Medicare data at the beneficiary level. This approach may partly reflect coding practices, not true differences in patient-level risk.^23,24^

## Conclusions

In this cross-sectional study of ACOs, we developed an external measure of ACO efficiency (ie, spending ratio), which varied considerably. We found that a higher ACO spending ratio, characterizing less efficient ACOs, was associated with greater odds of earning a bonus through the Shared Savings Program. The introduction of the regional benchmark adjustment afforded efficient ACOs more opportunities to earn bonuses. However, even after this policy change, it appears that the least efficient ACOs were still most likely to earn bonuses. Our study points to the continued need for benchmarking reform that can sustainably reward efficient ACOs, which is especially critical for voluntary programs.

## Notes

Supplement 1. eTable 1. Cohort data from Medicare claims used to calculate estimated spending by ACO quartile eTable 2. Patient-level characteristics pre- and post-2017 eTable 3. Adjusted odds of earning a shared savings bonus in second agreement year eTable 4. Adjusted odds of earning a shared savings bonus in third agreement year eTable 5. Adjusted odds of earning a shared savings bonus in fourth agreement year eFigure. Mean shared savings bonus per beneficiary by ACO quartile

Supplement 2. Data Sharing Statement
