# Supplementary material for: Accountable Care Organization Efficiency on Entry and Shared Savings Bonuses
Source: JAMA Netw Open. 2026 Feb 26;9(2):e260166. doi: 10.1001/jamanetworkopen.2026.0166 (PMC12947013; doi:10.1001/jamanetworkopen.2026.0166)
Supplement: Supplement 1. — eTable 1. Cohort data from Medicare claims used to calculate estimated spending by ACO quartile eTable 2. Patient-level characteristics pre- and post-2017 eTable 3. Adjusted odds of earning a shared savings bonus in second agreement year eTable 4. Adjusted odds of earning a shared savings bonus in third agreement year eTable 5. Adjusted odds of earning a shared savings bonus in fourth agreement year eFigure. Mean shared savings bonus per beneficiary by ACO quartile [file jamanetwopen-e260166-s001.pdf]

Supplemental Online Content

Srivastava A, Shay A, Kaufman SR, et al. Accountable care organization efficiency on entry and shared savings bonuses. *JAMA Netw Open.* 2026;9(2):e260166. doi:10.1001/jamanetworkopen.2026.0166

eTable 1. Cohort data from Medicare claims used to calculate estimated spending by ACO quartile

eTable 2A. Patient-Level characteristics pre- and post-2017,

ACO Quartiles 1 and 2

eTable 2B. Patient-Level characteristics pre- and post-2017,

ACO Quartiles 3 and 4

eTable 3. Adjusted Odds of Earning a Shared Savings Bonus in Second Agreement Year

eTable 4. Adjusted Odds of Earning a Shared Savings Bonus in Third Agreement Year

eTable 5. Adjusted Odds of Earning a Shared Savings Bonus in Fourth Agreement Year

eFigure: Mean shared savings bonus per beneficiary by ACO quartile

This supplemental material has been provided by the authors to give readers additional information about their work.

© 2026 Srivastava A et al. *JAMA Network Open*.

eTable 1: Cohort data from Medicare claims used to calculate estimated spending by ACO quartile

	Quartile 1	Quartile 2	Quartile 3	Quartile 4	p value
**Total Beneficiaries**	1325551	1480353	1496514	981923	
**Unique Beneficiaries**	613181	639558	673500	471554	
**Age, mean(SD)**	76.09 (7.68)	76.12 (7.68)	76.17 (7.78)	76.21 (7.74)	<0.001
**Female, n(%)**	771725 (58.22%)	862365 (58.25%)	877347 (58.63%)	573007 (58.36%)	<0.001
**Black, n(%)**	87998 (6.64%)	94220 (6.36%)	117987 (7.88%)	74275 (7.56%)	<0.001
**White, n(%)**	1134975 (85.62%)	1301529 (87.92%)	1253847 (83.78%)	793416 (80.8%)	<0.001
**ESRD, n(%)**	8574 (0.65%)	8382 (0.57%)	9448 (0.63%)	7479 (0.76%)	<0.001
**Disability, n(%)**	126312 (9.53%)	135987 (9.19%)	129836 (8.68%)	104293 (10.62%)	<0.001
**SES**					
**SES 0 , n(%)**	1180445 (89.05%)	1351325 (91.28%)	1353928 (90.47%)	836414 (85.18%)	<0.001
**SES 1 , n(%)**	78617 (5.93%)	66550 (4.5%)	80988 (5.41%)	87925 (8.95%)	
**SES 2 , n(%)**	66489 (5.02%)	62478 (4.22%)	61598 (4.12%)	57584 (5.86%)	
**HCC**					
**HCC 0 , n(%)**	408991 (30.85%)	452509 (30.57%)	450768 (30.12%)	270085 (27.51%)	<0.001
**HCC 1, n(%)**	338862 (25.56%)	376273 (25.42%)	377898 (25.25%)	242748 (24.72%)	
**HCC 2, n(%)**	221788 (16.73%)	248831 (16.81%)	254227 (16.99%)	169749 (17.29%)	
**HCC 3+, n(%)**	355910 (26.85%)	402740 (27.21%)	413621 (27.64%)	299341 (30.49%)	

ESRD = end stage renal disease, SES = socioeconomic status, HCC = Hierarchical Condition Category

© 2026 Srivastava A et al. *JAMA Network Open*.

eTable 2A: Patient-Level characteristics pre- and post-2017, ACO Quartiles 1 and 2

	ACO Quartile 1			*ACO Quartile 2*		
	**Pre**	**Post**	**p value**	**Pre**	**Post**	**p value**
**Total Beneficiaries, n**	686005	639546		827191	653162	
**Unique Beneficiaries, n**	395429	387053		433696	378781	
**Age, mean(SD)**	75.55 (7.86)	76.67 (7.44)	<0.001	75.74 (7.87)	76.61 (7.4)	<0.001
**Female, n(%)**	399486 (58.23%)	372239 (58.2%)	0.7259	482810 (58.37%)	379555 (58.11%)	0.002
**Black, n(%)**	45095 (6.57%)	42903 (6.71%)	0.0018	49721 (6.01%)	44499 (6.81%)	<0.001
**White, n(%)**	584434 (85.19%)	550541 (86.08%)	<0.001	727452 (87.94%)	574077 (87.89%)	0.35
**ESRD, n(%)**	4649 (0.68%)	3925 (0.61%)	<0.001	4733 (0.57%)	3649 (0.56%)	0.27
**Disability, n(%)**	64175 (9.35%)	62137 (9.72%)	<0.001	72382 (8.75%)	63605 (9.74%)	<0.001
**SES**						
**SES 0 , n(%)**	601075 (87.62%)	579370 (90.59%)	<0.001	749231 (90.58%)	602094 (92.18%)	<0.001
**SES 1 , n(%)**	46096 (6.72%)	32521 (5.09%)		41486 (5.02%)	25064 (3.84%)	
**SES 2 , n(%)**	38834 (5.66%)	27655 (4.32%)		36474 (4.41%)	26004 (3.98%)	
**HCC**						
**HCC 0 , n(%)**	230614 (33.62%)	178377 (27.89%)	<0.001	275876 (33.35%)	176633 (27.04%)	<0.001
**HCC 1, n(%)**	174480 (25.43%)	164382 (25.7%)		210511 (25.45%)	165762 (25.38%)	
**HCC 2, n(%)**	110835 (16.16%)	110953 (17.35%)		133985 (16.2%)	114846 (17.58%)	
**HCC 3+, n(%)**	170076 (24.79%)	185834 (29.06%)		206819 (25%)	195921 (30%)	

eTable 2B: Patient-Level characteristics pre- and post-2017, ACO Quartiles 3 and 4

	Quartile 3			*Quartile 4*		
	**Pre**	**Post**	**p value**	**Pre**	**Post**	**p value**
**Total Beneficiaries (n)**	954170	542344		534457	447466	
**Unique Beneficiaries (n)**	517454	376384		308264	271318	
**Age, mean(SD)**	75.85 (7.96)	76.73 (7.42)	<0.001	76 (7.96)	76.47 (7.47)	<0.001
**Female, n(%)**	560923 (58.79%)	316424 (58.34%)	<0.001	311724 (58.33%)	261283 (58.39%)	0.51
**Black, n(%)**	77920 (8.17%)	40067 (7.39%)	<0.001	39604 (7.41%)	34671 (7.75%)	<0.001
**White, n(%)**	794340 (83.25%)	459507 (84.73%)	<0.001	426703 (79.84%)	366713 (81.95%)	<0.001
**ESRD, n(%)**	6420 (0.67%)	3028 (0.56%)	<0.001	4159 (0.78%)	3320 (0.74%)	0.04
**Disability, n(%)**	78858 (8.26%)	50978 (9.4%)	<0.001	48674 (9.11%)	55619 (12.43%)	<0.001
**SES**						
**SES 0 , n(%)**	859042 (90.03%)	494886 (91.25%)	<0.001	455867 (85.3%)	380547 (85.04%)	<0.001
**SES 1 , n(%)**	53854 (5.64%)	27134 (5%)		48798 (9.13%)	39127 (8.74%)	
**SES 2 , n(%)**	41274 (4.33%)	20324 (3.75%)		29792 (5.57%)	27792 (6.21%)	
**HCC**						
**HCC 0 , n(%)**	306602 (32.13%)	144166 (26.58%)	<0.001	157155 (29.4%)	112930 (25.24%)	<0.001

© 2026 Srivastava A et al. *JAMA Network Open*.

**HCC 1, n(%)**	239982 (25.15%)	137916 (25.43%)	131569 (24.62%)	111179 (24.85%)
**HCC 2, n(%)**	157886 (16.55%)	96341 (17.76%)	90678 (16.97%)	79071 (17.67%)
**HCC 3+, n(%)**	249700 (26.17%)	163921 (30.22%)	155055 (29.01%)	144286 (32.25%)

ESRD = end stage renal disease, SES = socioeconomic status, HCC = Hierarchical Condition Category

eTable 3: Adjusted Odds of Earning a Shared Savings Bonus in Second Agreement Year

Variable		OR	95%CI		p value
First year ACO spending ratio	(0.01 increase)	1.20	1.03	1.39	0.02
% ESRD	1% increase	1.44	0.83	2.50	0.19
% Disability	1% increase	1.26	0.94	1.69	0.12
% Elderly	1% increase	1.28	0.97	1.69	0.09
% Non-white	1% increase	1.00	0.98	1.02	0.99

ESRD = end stage renal disease

eTable 4: Adjusted Odds of Earning a Shared Savings Bonus in Third Agreement Year

Variable		OR	95%CI		p value
First Year ACO spending ratio	(0.01 increase)	1.50	1.27	1.77	<0.001
% ESRD	1% increase	1.99	1.10	3.61	0.02
% Disability	1% increase	1.31	0.98	1.76	0.07
% Elderly	1% increase	1.29	0.98	1.71	0.07
% Non-white	1% increase	1.01	0.98	1.03	0.63

ESRD = end stage renal disease

eTable 5: Adjusted Odds of Earning a Shared Savings Bonus in Fourth Agreement Year

Variable		OR	95%CI		p value
First Year ACO spending ratio	(0.01 increase)	1.38	1.18	1.61	<0.001
% ESRD	1% increase	1.71	0.95	3.07	0.07
% Disability	1% increase	1.60	1.19	2.16	<0.001
% Elderly	1% increase	1.59	1.20	2.12	<0.001
% Non-white	1% increase	1.01	0.99	1.03	0.27

ESRD = end stage renal disease

© 2026 Srivastava A et al. *JAMA Network Open*.

eFigure: Mean shared savings bonus per beneficiary by ACO quartile

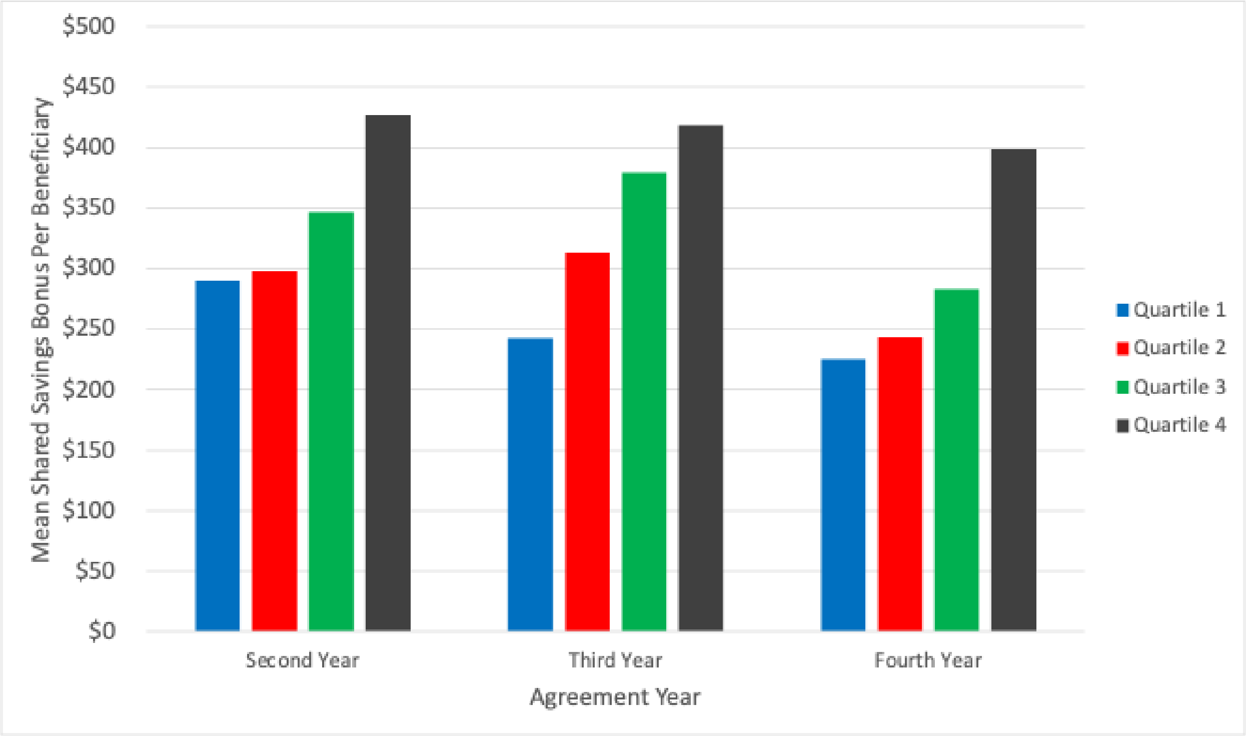

© 2026 Srivastava A et al. *JAMA Network Open*.
