# Supplementary material for: Accountable Care Organization Efficiency on Entry and Shared Savings Bonuses
Source: JAMA Netw Open. 2026 Feb 26;9(2):e260166. doi: 10.1001/jamanetworkopen.2026.0166 (PMC12947013; doi:10.1001/jamanetworkopen.2026.0166)
Supplement: Supplement 2. — Data Sharing Statement [file jamanetwopen-e260166-s002.pdf]

Data Sharing Statement

Srivastava. Accountable Care Organization Efficiency on Entry and Shared Savings Bonuses. *JAMA Netw Open*. Published February 26, 2026. doi:10.1001/jamanetworkopen.2026.0166

Data
Data available: No

Additional Information
Explanation for why data not available: The data underlying this article were provided by the Centers for Medicare and Medicaid Services and cannot be shared by the authors of this manuscript under the data use agreement.
